# Editorial: Drug repurposing and polypharmacology: A synergistic approach in multi-target based drug discovery

**DOI:** 10.3389/fphar.2022.1101007

**Published:** 2022-12-20

**Authors:** Mithun Rudrapal, Keshav Raj Paudel, Rudra Pangeni

**Affiliations:** ^1^ Department of Pharmaceutical Chemistry, Rasiklal M. Dhariwal Institute of Pharmaceutical Education & Research, Pune, India; ^2^ Department of Oriental Medicine Resources, Mokpo National University, Muan-gun, South Korea; ^3^ Virginia Commonwealth University, Richmond, VA, United States

**Keywords:** drug repurposing, polypharmacology, multi-targeting, drug discovery, cancer, COVID-19

Drug repurposing (also called drug repositioning) is a process of identifying new therapeutic uses for approved and/or existing drugs for treating common, difficult-to-treat and rare diseases (Paul et al., 2022; Rudrapal et al., 2022). On the other hand, polypharmacology (or multi-targeting approach) involves the interactions of drug molecules with multiple targets of different therapeutic indications/diseases (Jamir et al., 2022).

Drug repurposing is increasingly becoming an attractive strategy worldwide as it involves lower risk, potentially reduced expenditure and shorter development timelines as compared to *de novo* drug discovery (Rudrapal et al., 2020). Rising scenarios of deadly diseases (cancer, cardiovascular illness, diabetes, infectious diseases, COVID-19) largely affect the lives of millions of people, and thereby it impose a heavy economic burden globally (Singh et al., 2020). Currently available (or FDA approved) drugs are inadequate to manage a majority of such diseases, and, therefore, there is an urgent need for new drug candidates and/or drug therapy. Drugs with multi-targeting (polypharmacology approach) potential are immensely interesting in repurposing, because this dual synergistic strategy could offer better therapeutic alternative and useful clinical candidates (Pinzi et al., 2021).

The Research Topic *“Drug repurposing and polypharmacology: A synergistic approach in multi-target based drug discovery”* was aimed to compile latest research ideas, directions, developments and advances focusing on the theme of the topic within the scope of the journal. The topic was led by three Guest Editors listed above who are experts in the subject and oversaw the entire editorial process for the submitted papers. A total of ten articles were published, including seven original research and three review articles.

In a study, Xie et al. reported that lenvatinib when combined with the PD-1 inhibitor could effectively treat patients with advanced intrahepatic cholangiocarcinoma (ICC). They concluded that this combination therapy could be a safe and better alternative option for the treatment of advanced ICC.

A review article by Liu et al. highlighted the development of novel antiviral compounds targeting the S protein of SARS-CoV-2 through screening of natural products and drug repurposing approaches. This study provided insights into the discovery of promising drug candidates from natural sources as possible anti-SARS-CoV-2 agents.

Another study reported by Yang et al. utilized a molecular docking protocol to screen out potential inhibitors targeting the main protease (M^pro^) of SARS-CoV-2. This study resulted in five compounds (namely, N-1H-Indazol-5-yl-2-(6-methylpyridin-2-yl)quinazolin-4- amine, ergotamine, antrafenine, dihydroergotamine and phthalocyanine) as potential drug candidates to be developed for clinical trials. Further, molecular dynamics (MD) simulations confirmed that potential inhibitory effect of the five identified compounds against SARS-CoV-2 M^pro^.

Mangione et al. investigated upon Computational Analysis of Novel Drug Opportunities (CANDO) platform to identify small molecule inhibitors against COVID-19 on the basis of multiscale therapeutic, repurposing and design approaches. Interestingly, 51 of their 276 predictions demonstrated anti-SARS-CoV-2 potential according to published reports (clinical and experimental), suggesting the ability of CANDO platform in multi-target based drug discovery.

In another study, Khan et al. investigated molecular targets and pathways of nitazoxanide as novel approaches for the treatment of hepatocellular carcinoma (HCC) by using molecular docking and network pharmacology approaches. Authors proposed that distinct therapeutic effect for nitazoxanide is possible in treating HCC, with well-defined pharmacological targets and molecular pathways.

Sun et al. represented a bibliometric analysis of publications on drug repurposing for 10 years (2010–2020), which included 2,978 of publications. Their findings reported that the United States leads in drug repurposing research, followed by China, the United Kingdom, and India. From keyword analysis, they also reported that the hotspots have been changed in recent years, with COVID-19/SARS-CoV-2/coronavirus being the most prominent topic(s) in the domain of drug discovery.

A study by Kusuma et al. proposed an approach to implement bipartite graph search optimization using the branch and bound algorithm to identify the combination or composition of Jamu formulas. In addition, the proposed method comprising one to four selected plant species for the T2DM Jamu formula was suggested by the researchers.

Cao et al. predicted the mechanism of action of licorice in the treatment of COVID-19 through an extensive computational analysis using bioinformatics tools and molecular dynamics simulation. Authors reported that phytochemicals (phaseol, glycyrol, glyasperin F) present in licorice could act against COVID-19 through the inhibition of STAT3, IL2RA, MMP1 and CXCL8.

Wang et al. investigated the target-specific compound selectivity for multi-target drug discovery and repurposing by experimental studies. Authors represented several case studies exhibiting target-specific selectivity, which could facilitate the repurposing drugs by multi-targeting approach.

A review article by Kakoti et al. summarized therapeutic drug repositioning approaches for neurodegenerative diseases with recent threats and issues. They demonstrated the neuroprotective effect of kinase inhibitors, which, however, were originally developed for oncological indications. Authors also highlighted several opportunities and challenges of drug repurposing approaches in the way of drug discovery despites many technological advancements.

In conclusion, this Research Topic has provided in-depth insights into newer research findings (experimental, computational and review reports) and latest updates including technological advancements and challenges) related to ongoing repurposing strategies and drug discovery research in various therapeutic areas of current interest. Though drug repurposing strategies have several potentials as already indicated above, it has many challenges in the process of drug discovery, whether from a scientific or regulatory perspectives. Critical evaluations of pre-clinical, clinical and observational data/evidences are required to investigate the therapeutic efficacy and safety/toxicity of a candidate drug for potential repurposing.
